# Mutation in *MSH5* Causes Primary Ovarian Insufficiency and Successful Therapeutic Intervention by In Vitro Fertilisation

**DOI:** 10.1111/jcmm.70745

**Published:** 2026-05-08

**Authors:** Bin Mao, Lili Zhang, Xiaodong Zhao, Xiaojuan Xu, Hongfang Liu, Dexiao Song, Peiqiang Li, Santasree Banerjee, Xiaoling Ma, Yongxiu Yang

**Affiliations:** ^1^ The First School of Clinical Medicine Lanzhou University Lanzhou Gansu China; ^2^ The First Hospital of Lanzhou University Lanzhou Gansu China; ^3^ Institute of Genetics, School of Basic Medical Sciences, Lanzhou University Lanzhou China; ^4^ Department of Genetics, College of Basic Medical Sciences Jilin University Changchun Jilin China

**Keywords:** *MSH5* gene, novel variant, primary ovarian insufficiency, splice‐donor site, whole exome sequencing

## Abstract

Primary ovarian insufficiency (POI) is a genetically heterogeneous disorder characterised by cessation of menstruation before the age of 40 years with elevated levels of follicle‐stimulating hormone. Germline variants in the *MSH5* gene cause POI. In this study, we investigated a Han Chinese family with POI. The proband and her two younger sisters are clinically diagnosed with POI. The proband's parents are phenotypically normal. Whole exome sequencing identified a novel homozygous splice‐donor site (c.271+1G>A) variant in the first nucleotide of intron 3 of the *MSH5* gene in this proband. Sanger sequencing confirmed that both the younger sisters of the proband also harbour the same homozygous variant, while the proband's parents are carrying this variant in a heterozygous state. This variant causes aberrant splicing of MSH5 mRNA followed by the formation of an alternative MSH5 transcript with complete loss of exon 3. Relative expression of mutant MSH5 mRNA is significantly reduced compared to that of the wild‐type transcript. Mutant MSH5 impaired DNA homologous recombination repair as well as significantly lowering the clonogenic survival rate of cell clone formation compared with the wild type. In addition, we also found a significantly higher apoptotic rate and lower cell proliferation rate in the mutant than that of the wild type. We performed egg donation‐based in vitro fertilisation for this proband, and two healthy baby girls have been successfully delivered. Our present study reports the first splice‐site variant in the *MSH5* gene in patients with POI. We also report for the first time the successful therapeutic intervention by in vitro fertilisation for patients with *MSH5‐associated* POI.

## 1 Introduction

Primary ovarian insufficiency (POI) [MIM# 311360] is a hypergonadotropic disorder characterised by cessation of menstruation before the age of 40 years with elevated levels of follicle stimulating hormone (FSH) [1, 2]. POI is globally affecting 1% of women before the age of 40, 0.01% of women before the age of 30, and 5% of women prior to the age of 45 with extreme genotypic and phenotypic heterogeneity [3, 4]. However, POI is presented in both syndromic and non‐syndromic forms [5]. In addition, patients with POI manifest premature loss of ovarian reserve, followed by primary or secondary amenorrhoea and hypergonadotropic hypogonadism which finally leads to infertility [6]. POI patients are clinically diagnosed by elevated levels of gonadotropin, hypoestrogenism, and amenorrhea with menopausal levels of FSH, estradiol (E2), and anti‐mullerian hormone (AMH) [7]. POI patients are identified with extreme phenotypic heterogeneity, from milder forms with defective folliculogenesis and secondary amenorrhea to severe forms with absence of pubertal development and primary amenorrhea [8].

The aetiology of POI is also highly heterogeneous, majorly including structural abnormalities of chromosomes and genetic variants [9]. Additionally, more than half of the POI cases remain idiopathic with both sporadic and familial forms of presentation [10]. Although isolated POI often appears sporadically, the presence of affected first‐degree relatives in POI cases strongly indicates the involvement of significant genetic aetiology [10, 11]. Moreover, genetic factors underlying POI have been reported to be identified in 20%–25% of POI cases [9, 11, 12]. It has long been reported that chromosomal abnormalities are also associated with 10%–15% of POI cases [13]. It has also been well investigated and found that germline variations in candidate genes associated with POI aetiology are significantly involved in DNA repair, meiosis, transcriptional regulation, gonadal development, and hormonal signalling [14, 15]. Recently, whole exome sequencing identified novel candidate genes, namely, *MSH5*, *HFM1*, *STAG3*, *MCM8*, *MCM9*, and *CSB‐PGBD3* associated with POI aetiology [16]. Significantly, all of these POI‐associated candidate genes play a key role in DNA mismatch repair or meiosis, which allows us to understand the association between the pathogenesis of POI and DNA mismatch repair [17].

*MSH5* gene is located in the short arm of chromosome 6 (6p21.33) and contains 25 exons (NM_025259). *MSH5* gene encodes MSH5 (MutS homologue 5) protein of 834 amino acids [18]. MSH5 is one of the important members of the DNA mismatch repair (MMR) protein family, involved in DNA mismatch repair by correcting mispaired bases due to erroneous DNA replication [16, 18]. Although MSH5 protein is widely expressed, but it shows a high expression level in testis and ovary, specifically in granulosa cells [19]. However, the expression of MSH5 protein is also found in fetal ovary and adrenal gland [16]. In addition, as like as other MMR protein family members, MSH5 protein specifically binds with MSH4 protein and leads to the formation of MSH4‐MSH5 heterodimers [20]. MSH4‐MSH5 heterodimers comprise an ATPase domain, promoting the exchange of ADP to ATP followed by the formation of a sliding clamp for encircling DNA to perform homologous recombination (HR) repair for DNA double strand breaks (DSBs) [21, 22]. MSH4‐MSH5 heterodimers play a key role in DNA mismatch repair and meiotic recombination processes as well as stabilising and protecting the meiotic recombination intermediate [14]. During meiosis, MSH4‐MSH5 heterodimers are also found to facilitate chromosomal synapsis [20]. Therefore, germline variants in either *MSH5* or *MSH4* cause impairment of the formation of MSH4‐MSH5 heterodimers, leading to the failure of homologous synapsis followed by cell death before the pachytene stage of prophase I during meiosis, which finally results in infertility in humans and mice [19, 23].

In this study, we investigated a nonconsanguineous Han Chinese family clinically diagnosed with POI. The proband and her two younger sisters are clinically diagnosed with POI. The proband's parents are phenotypically normal. In order to identify the disease‐causing variant, we performed whole exome sequencing and confirmatory Sanger sequencing. Whole exome sequencing identified a novel homozygous splice‐donor site (c.271+1G>A) variant in the *MSH5* gene in this proband. Sanger sequencing confirmed that this homozygous novel variant co‐segregates well with the disease phenotype among the affected members of this family. Then, we functionally characterise the identified variant to understand the disease mechanism. Our present study reports the first splice‐site variant in the *MSH5* gene in patients with POI.

## 2 Materials and Methods

### 2.1 Patients and Families

**FIGURE 1:**
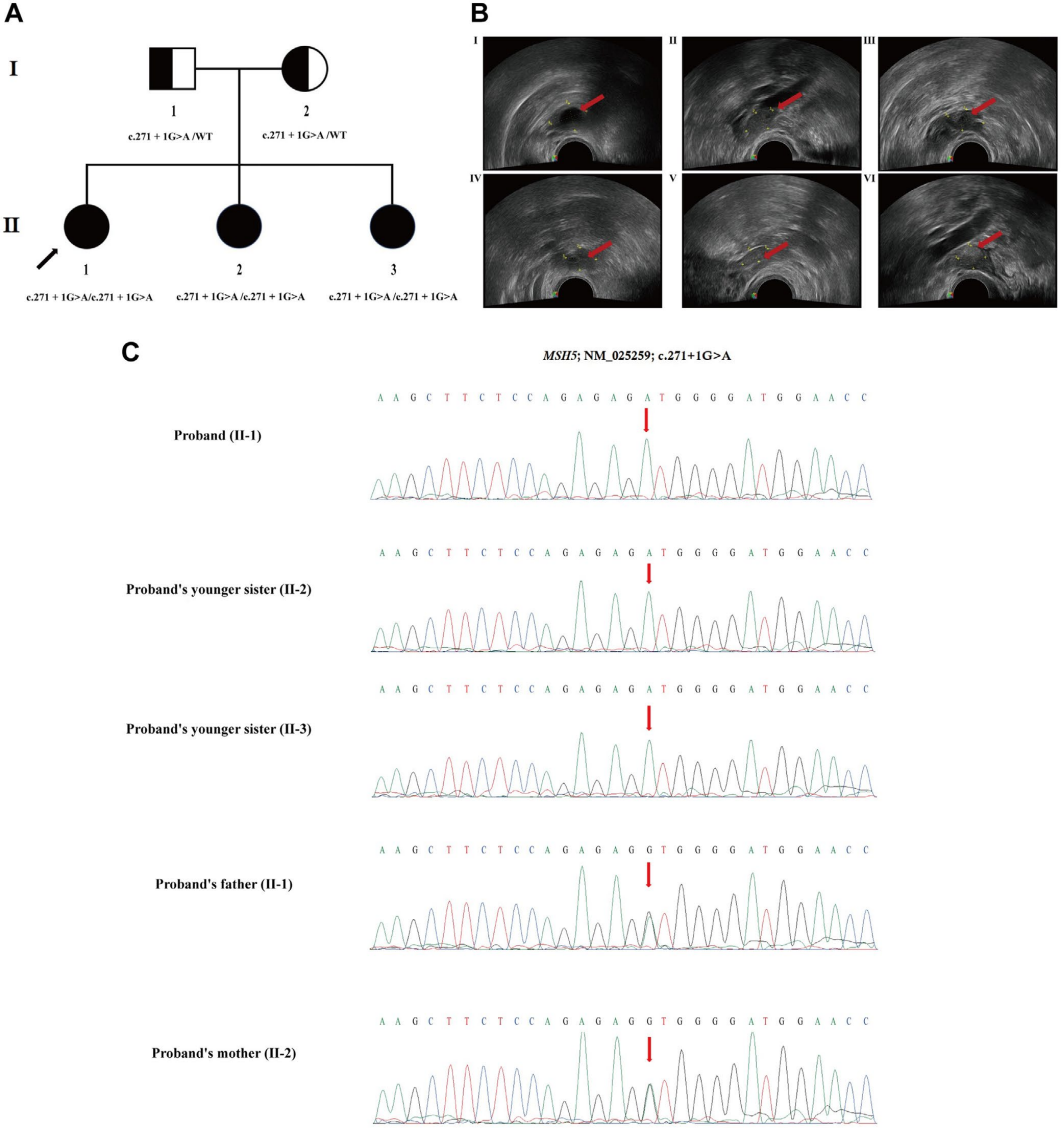
(A) Pedigree of family. Pedigree of the described nonconsanguineous Chinese family with POI. Squares and circles denoted males and females respectively. Individuals labelled with a solidus were deceased. Roman numerals indicate generations. Arrow indicates the proband (II‐1). (B) Transvaginal B‐ultrasonography. B‐ultrasound examination of proband (II‐1), proband's younger sister (II‐2) and proband's youngest sister (II‐3). The “red arrow” is showing the ovary. (I): The left ovary of the proband (II‐1) is 21.56*15.81 mm. (II): The right ovary of the proband (II‐1) is 19.51*11.35 mm. (III): The left ovary of the proband's younger sister (II‐2) is 20.74*12.22 mm. (IV): The right ovary of the proband's younger sister (II‐2) is 17.55*12.88 mm. (V): The left ovary of the proband's youngest sister (II‐3) is 17.08*8.06 mm. (VI): The right ovary of the proband's youngest sister (II‐3) is 15.75*13.27 mm. (C) Sanger sequencing. Partial DNA sequences in the MSH5 gene by Sanger sequencing of the family. The reference sequence NM_025259 of MSH5 gene was used.

Here, a Han Chinese family with POI was enrolled on January 3rd, 2023, in The First School of Clinical Medicine, Lanzhou University, Lanzhou, 730,000, China (Figure 1A). The study was approved by the ethics committee of The First School of Clinical Medicine, Lanzhou University, Lanzhou, 730,000, China, in accordance with the recommendations of the Declaration of Helsinki. We obtained written informed consent from all the participants for this study.

### 2.2 Whole Exome Sequencing

Whole exome sequencing (WES) was performed for the proband. We collected the proband's peripheral blood, extracted genomic DNA (QIAamp DNA Micro Kit, Catalogue no./ID: 56304, QIAGEN Inc., US) and performed WES [24, 25]. Firstly, we prepared the sequencing library (Illumina DNA Prep with Exome 2.5 Enrichment, Catalogue no./ID: 20077595, Illumina Inc., US) by capturing exome sequences with Agilent SureSelect version 8 (Agilent Technologies, Santa Clara, CA, US). Next, the enriched sequencing library was subjected to WES by Illumina NovoSeq 6000. Then, Burrows‐Wheeler Aligner software (http://bio‐bwa.sourceforge.net, version 0.59) was used to align the WES obtained sequencing reads with GRCh37.p10. After that, GATK IndelRealigner (https://gatk.broadinstitute.org) was used to locally realign the Burrows‐Wheeler aligned reads. Next, the base quality recalibration was performed with the Burrows‐Wheeler aligned sequencing reads by using GATK Base Recalibrator (https://gatk.broadinstitute.org). Then, single‐nucleotide variants (SNVs) and insertions or deletions (InDels) were identified by using GATK Unified Genotyper (https://gatk.broadinstitute.org). After that, all these identified variants (both SNVs and InDels) were annotated with the consensus coding sequence database (20,130,630) of the National Center for Biotechnology Information (NCBI; https://www.ncbi.nlm.nih.gov/CCDS/CcdsBrowse.cgi). Image analysis and base calling were performed through using the Illumina pipeline. We designed indexed primers for data fidelity surveillance. SOAP aligner (soap2.21) (http://soap.genomics.org.cn) software was used to align the clean sequencing reads with the human reference (hg19, https://www.ncbi.nlm.nih.gov/datasets/genome). Finally, we assembled the consensus sequences and used SOAPsnp (http://soap.genomics.org.cn, v1.05) software for calling genotype. Quality control data of whole exome sequencing for the proband (II‐1) have been presented in Table S1.

### 2.3 Bioinformatics Data Analysis and Interpretation

All these variants obtained by WES were collected. We selected variants for bioinformatic data analysis and interpretation if their minor allele frequency (MAF) is < 0.01 in the following public databases: dbSNP (https://www.ncbi.nlm.nih.gov), 1000 Genome Database (http://www.internationalgenome.org), HapMap (https://www.genome.gov), and our in‐house database of 50,000 Han Chinese samples [26, 27, 28]. Firstly, we compared our identified variants with the Human Gene Mutation Database (HGMD, https://www.hgmd.cf.ac.uk/) to understand whether the identified genetic variants are a novel variant or previously reported to be associated with any monogenic diseases [29]. Then, we compared our identified variants with the Online Mendelian Inheritance in Man (OMIM, https://www.omim.org) database to understand the genotype–phenotype correlation and inheritance pattern of our identified genes and their variants [30]. Next, we compared our identified variants with the Exome Aggregation Consortium (ExAC, https://exac.broadinstitute.org) database to confirm whether our identified genetic variations were already reported in some healthy individuals from different populations [31]. After that, we compared our identified genetic variations with the Genome Aggregation Database (gnomAD, https://gnomad.broadinstitute.org) to understand the frequency of the identified genetic variations in different populations [32].

Then, we interpreted our identified genetic variations according to the variant interpretation guidelines of the American College of Medical Genetics and Genomics (ACMG) [33]. Next, we used *in silico* webservers, namely, SIFT (http://provean.jcvi.org/index.php), Polyphen‐2 (http://genetics.bwh.harvard.edu/pph2), MutationTaster (http://doro.charite.de/MutationTaster), and REVEL (https://genome.ucsc.edu/cgibin/hgTrackUi?db=hg19&g=revel), to predict the possible pathogenic impacts of our identified genetic variations. Splice‐AI (https://spliceailookup.broadinstitute.org/) was employed to evaluate the ability of our identified genetic variations to disrupt splice sites. Finally, we selected the most potentially pathogenic genetic variation underlying the disease phenotype in this case. In addition, “Mutalyzer 2” (https://v2.mutalyzer.nl/) software was used to confirm the expression of the variant according to the Human Genome Variation Society (HGVS) nomenclature guideline [34].

### 2.4 Sanger Sequencing

We performed Sanger sequencing to validate the hemizygous novel missense variation identified by WES. Primers (F1 5′‐AGGCGATGCGGCGGTTTCTAGC‐3′, R1 5′‐GGCCGCGGCGATCGGCCGTG‐3′) were designed according to the reference (NM_025259 of *MSH5*) genomic sequences of the Human Genome from GenBank in the National Center for Biotechnology Information (NCBI; https://www.ncbi.nlm.nih.gov/genbank/). Sanger sequencing was performed with the PCR products, and data were compared and analysed.

### 2.5 Reverse Transcription Polymerase Chain Reaction (RT‐PCR) and cDNA Sequencing

Reverse transcription polymerase chain reaction (RT‐PCR) and cDNA sequencing have been performed to understand the effect of this homozygous novel splice‐donor site variant of the *MSH5* gene on the splicing event of MSH5 mRNA. We isolated and extracted total RNA from the peripheral blood samples of all the family members by using the Tiangen blood RNA extraction kit (DP402). Then, we performed RT‐PCR and obtained cDNA from the total RNA according to the manufacturer's protocol (RevertAid First Strand cDNA Synthesis Kit, Thermo Scientific, K1622). After that, primers (F‐5′‐TTAGGAGCGAACCCAAGGAG‐3′, R‐5′‐TCCAGAAAGGAGGCGTTGTT‐3′) were designed for encompassing the coding sequence from exon 2 to exon 5. These primers were used to amplify the cDNA, and PCR fragments were recovered and subjected to sequencing followed by data analysis.

### 2.6 Analysis of *MSH5* Gene Expression Level by Quantitative Real Time PCR (qPCR)

Quantitative Real Time PCR (q‐PCR) was performed to identify the relative expression of wild type and mutated MSH5 mRNA in the proband and all of her family members. cDNAs of the proband and her family members were subjected to fluorescence quantitative detection by quantitative Real Time PCR (q‐PCR) by using primers (F‐5′‐ACGAGTGCCAAACAGGATGA‐3′ and R‐5′‐GTCATGGCGTCTGGGATGAA‐3′). The q‐PCR was performed with both the target gene and housekeeping gene (GAPDH) for each of the samples (GAPDH‐F‐5′‐TCTGACTTCAACAGCGACACC‐3′, GAPDH‐R‐5′‐CTGTTGCTGTAGCCAAATTCGT‐3′). Relative expression of both wild type and mutated MSH5 mRNAs has been analysed by calculating the detected delta–delta cycle threshold (2^−∆∆CT^) value.

### 2.7 DNA Damage and Recovery Assay

The human osteosarcoma U2OS cells were transiently transfected with wild type or mutant MSH5‐GFP and MSH5‐Flag plasmids, and were incubated in media containing Etoposide (ETO) (5 μg/mL) for 1 h at 37°C to induce DSBs. Etoposide is a broadly employed eukaryotic topoisomerase II poison that stabilises cleaved DNA intermediates to promote DNA breakage. Then, the normal media was replaced, followed by recovery for 2 h at 37°C. Phosphorylation of the Ser‐139 residue of histone variant bH2AX leads to the formation of γ‐H2AX. γ‐H2AX is an early cellular response to the induction of DSBs. Detection of this phosphorylation event has emerged as a highly specific and sensitive molecular marker for monitoring DNA damage initiation and resolution. γ‐H2AX was used as a sensitive marker for DSBs in this study.

We created five groups for studies that were as follows: Control (without ETO), MSH5‐OE (replace the normal medium after treatment with wild type MSH5 with ETO for 1 h), MSH5‐OE‐1 (change the normal medium after treatment of wild type MSH5 with ETO for 1 h and leave it for 2 h), MSH5‐OE‐2 (replace the normal medium after treatment of mutant MSH5 with ETO for 1 h), MSH5‐OE‐3 (replace the normal medium after treatment of mutant MSH5 with ETO for 1 h and leave it for 2 h).

Cellular immunofluorescence was performed with the cells in well‐growing condition after plasmid transfection and ETO treatment by using both primary (GFP + γ‐H2AX 1:200) and secondary antibodies. Cells were observed under a fluorescence microscope (Motic, BA410E).

In order to detect the expression of the target protein, western blot was performed by extracting the protein from cells in well‐growing condition after plasmid transfection and ETO treatment. Primary antibodies used in western blot are as follows: Flag (Abcam ab1162, 1:3000), P‐H2AX (Santa Cruz SC‐517348, 1:100), GAPDH (Abcam ab181602, 1:10000). Secondary antibodies used in the western blot experiment are as follows: Goat anti‐Mouse IgG (H + L) Secondary antibody (Thermo Pierce Item No: 31160; 1:5000) and Goat anti‐Rabbit IgG (H + L) Secondary antibody (Thermo Pierce Item No: 31210, 1:5000). Using SuperSignal West Dura Extended Duration Substrate (Thermo Pierce, Item No: 34075) and ECL reagent (ECL DualVue WB Marker, GE, Item No: RPN810) was used to develop the X‐ray film (Huadong Medicine, China). Image J software was used to analyse the optical density value of each of these bands. Cellular immunofluorescence and western blot were performed to detect DNA damage and recovery by staining γ‐H2AX.

### 2.8 Cell Clone Formation Assay

Cell clone formation is one of the effective methods for measuring cell proliferation ability. Clone formation rate has been counted to quantitatively analyse the proliferation ability and independent survival ability of cells. The clonogenic survival rate was calculated in U2OS cells after ETO treatment, in which endogenous MSH5 was silenced with siRNA and then wild type or mutant MSH5 was overexpressed.

Cells in each experimental group were trypsinised and resuspended in complete medium. Inoculate 400–1000 cells/well in each experimental group in a 6‐well culture plate, and culture the inoculated cells in the incubator for 2 weeks or until the number of cells in most single clones is > 50. Then, cell clones were observed under a fluorescence microscope (Mshot, MF53). Next, we added 1000 μL of crystal violet staining solution to stain the cells and photographed them with a digital camera to count the clones.

### 2.9 Cellular Apoptosis Assay

The association between the target gene or the effect of drugs and cell apoptosis can be tested by detecting the number of cells in an apoptotic state by flow cytometry with annexin V‐APC single staining method.

Cells of each experimental group were trypsinized and resuspended them to form a cell suspension. Supernatant cells were collected (cell number ≥ 5 × 10^5^) and centrifuged to collect the cell pellet. Cell pellets were again centrifuged and resuspended in 200 μL 1 × binding buffer. Finally, we added 2 μL Annexin V‐APC at room temperature for 20–60 min. According to the number of cells, add 200–300 μL 1 × binding buffer and observe under a fluorescence microscope (Mshot, MF53).

### 2.10 Cell Counting Kit‐8 (CCK8) Assay

Cell Counting Kit‐8 (CCK8) assay was performed to understand the effect of the target gene or the effect of drugs on cell proliferation by detecting cell viability.

Cells of each group were trypsinized, centrifuged, and cell pellets were resuspended. Then, 2.5 × 10^4^ cells from the cell suspension were collected and added to complete medium. Next, we made five 96‐well plates with 100 μL cell suspension/well and five replicate wells per plate. On the second day after plating, select a 96‐well plate and add 10 μL CCK‐8 solution [2‐(2‐methoxy‐4‐nitrophenyl)‐3‐(4‐nitrophenyl)‐5‐(2,4‐disulfonic acid benzene)‐2H‐tetrazole monosodium salt] to each well. Incubate the culture plate in the incubator for 2 h. Measure the absorbance at 450 nm with a microplate reader. Test for five consecutive days, add CCK‐8 at the same time point every day, and incubate for the same time in the incubator.

## 3 Results

### 3.1 Human Subjects

Here, we investigated a Han Chinese family with POI (Figure 1A). Proband (II‐1), her younger sister (II‐2), and her youngest sister (II‐3) are from a nonconsanguineous family. Proband and her two sisters have been clinically diagnosed with oligomenorrhea since their menarche and later with amenorrhea with elevated levels of serum FSH, infantile uteri, and atrophic ovaries with devoid of follicles. Proband and her younger sisters have not been reported with chromosomal abnormalities, FMR1 premutation, autoimmune disorders, previous ovarian surgery, or chemo or radiotherapy.

Clinical description of the proband (II‐1), proband's younger sister (II‐2), and proband's youngest sister (II‐3) has been presented in Table S2. Transvaginal B‐ultrasonography of the proband (II‐1), her younger sister (II‐2), and her youngest sister (II‐3) has been shown in Figure 1B I–VI.

Proband's mother (I‐2) is a 60‐year‐old Han Chinese woman. She has not been identified with any clinical symptoms. She has menopause at the age of 47.

The proband (II‐1) was married and her husband's semen is normal. During her first and second pregnancies, she had a spontaneous abortion for more than 30 days of pregnancy. After that, no contraception or pregnancy was reported. The proband's younger sister (II‐2) was also married and her husband's semen is also normal. However, she has not yet been reported with contraception and pregnancy. The proband's youngest sister (II‐3) was also married and her husband's semen is also normal. Moreover, she has not yet been reported with contraception and pregnancy. The proband and her two sisters were clinically diagnosed with POI according to their significantly lower level of AMH (< 1 ng/mL) as well as reduced ovarian volume (< 40.00*30.00 mm) and decreased number of basal antral follicles (2–10 mm, < 5) than those of normal women in this age group.

### 3.2 Treatment

We collected four donated normal eggs which were subsequently fertilised by in vitro fertilisation (IVF). Then, two third‐day (D3) embryos were obtained and cryopreserved. After 5 months, four embryos were thawed, continued to grow overnight, and were transferred successfully, and the proband obtained twin pregnancies. Finally, two healthy baby girls were delivered at full term.

### 3.3 Identification of a Homozygous Novel Splice‐Donor Site Variant in *MSH5* Gene

WES identified a novel homozygous splice‐donor site variant (c.271+1G>A) in the intron 3 of the *MSH5* gene in the proband. Sanger sequencing confirmed that both the younger sisters of the proband were also carrying the same homozygous variant. The father and mother of the proband were harbouring this variant in a heterozygous state (Figure 1C).

This variant is also not present in the Human Gene Variant database (HGMD, www.hgmd.cf.ac.uk/), Online Mendelian Inheritance in Man (MIM, https://www.omim.org). This homozygous novel variant is also not found in our in‐house database of ~50,000 Chinese Han samples. We also did not find this variant in ExAC (https://exac.broadinstitute.org), gnomAD (https://gnomad.broadinstitute.org), dbSNP (https://www.ncbi.nlm.nih.gov/SNP), and 1000 Genome Database (https://www.internationalgenome.org).

According to the variant interpretation guidelines of the American College of Medical Genetics and Genomics (ACMG), this novel homozygous splice‐donor site mutation (c.271+1G>A) was classified as “*likely pathogenic*” variants [33].

Furthermore, this novel homozygous splice‐donor site variant (c.647+1G>A) in the *MSH5* gene was co‐segregated well with the disease phenotype in this family with an autosomal recessive mode of inheritance.

### 3.4 Functional Characterisation of Splice‐Donor Site Variant in *MSH5* Gene

**FIGURE 2:**
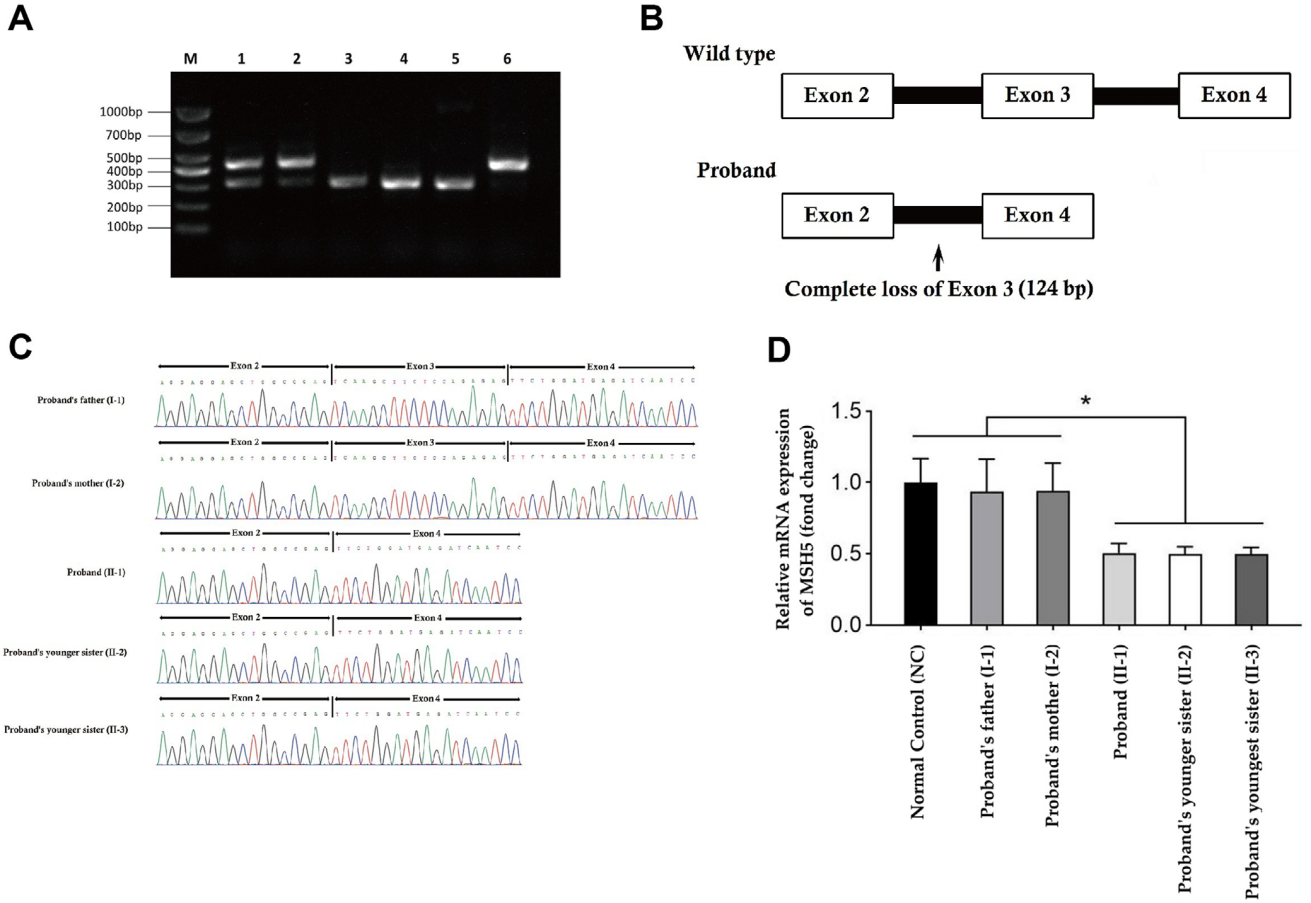
Reverse transcription polymerase chain reaction (RT‐PCR) and cDNA sequencing. (A) Agarose gel electrophoresis. M: 2000 bp marker; 1. Proband's father; 2. Proband's mother; 3. Proband; 4. Proband's younger sister; 5. Proband's younger sister; 6. Normal control product. Proband's father and mother are showing two electrophoretic bands of 428 bp and 304 bp, while the proband and both of her sisters are showing only one electrophoretic band of 304 bp. (B) cDNA sequencing. Both of these 428 bp and 304 bp bands were recovered to perform Sanger sequencing and the sequencing results were analysed. The longer RT‐PCR product is 428 bp in length and consisting of the sequences of exon 2, exon 3, exon 4 and exon 5, while the shorter RTPCR product is 304 bp in length and containing the sequences of exon2, exon 4, exon 5. (C) Schematic presentation of splicing event. Normal splicing event was found in both proband's father and mother. Aberrant splicing event was identified in proband and her two younger sisters with complete loss of exon 3. (D) Quantitative real‐time PCR (qPCR). Relative quantitative of MSH5 gene between different samples (*p < 0.05). The relative expression of mutant MSH5 transcript in proband and her two sisters is significantly reduced (*p* < 0.05) than the wild type MSH5 transcript in the proband's parents as well as in normal healthy control.

The RT‐PCR product of proband's father and mother produced two electrophoretic bands (one longer and one shorter product), while the RT‐PCR product obtained from the proband and both of her sisters produced only one electrophoretic band (only the shorter product) (Figure 2A). Both of these longer and shorter RT‐PCR products were recovered to perform Sanger sequencing, and the sequencing results were analysed. The longer RT‐PCR product is 428 bp in length and consists of the sequences of exon 2, exon 3, exon 4, and exon 5, while the shorter RT‐PCR product is 304 bp in length and contains the sequences of exon 2, exon 4, and exon 5 (Figure 2B). Hence, the homozygous novel splice‐site (c. 271+1G>A) variant causes a complete loss of exon 3 in the proband and her two sisters. The splicing event has been schematically presented (Figure 2C). Proband's parents are carrying the wild type transcript as well as the mutant MSH5 transcript.

### 3.5 Analysis of *MSH5* Gene Expression Level by qPCR

The relative expression of mutant MSH5 transcript in proband and her two sisters is significantly reduced (*p* < 0.05) compared to the wild type MSH5 transcript in the proband's parents as well as in normal healthy control (Figure 2D).

### 3.6 DNA Damage and Recovery Assay

**FIGURE 3:**
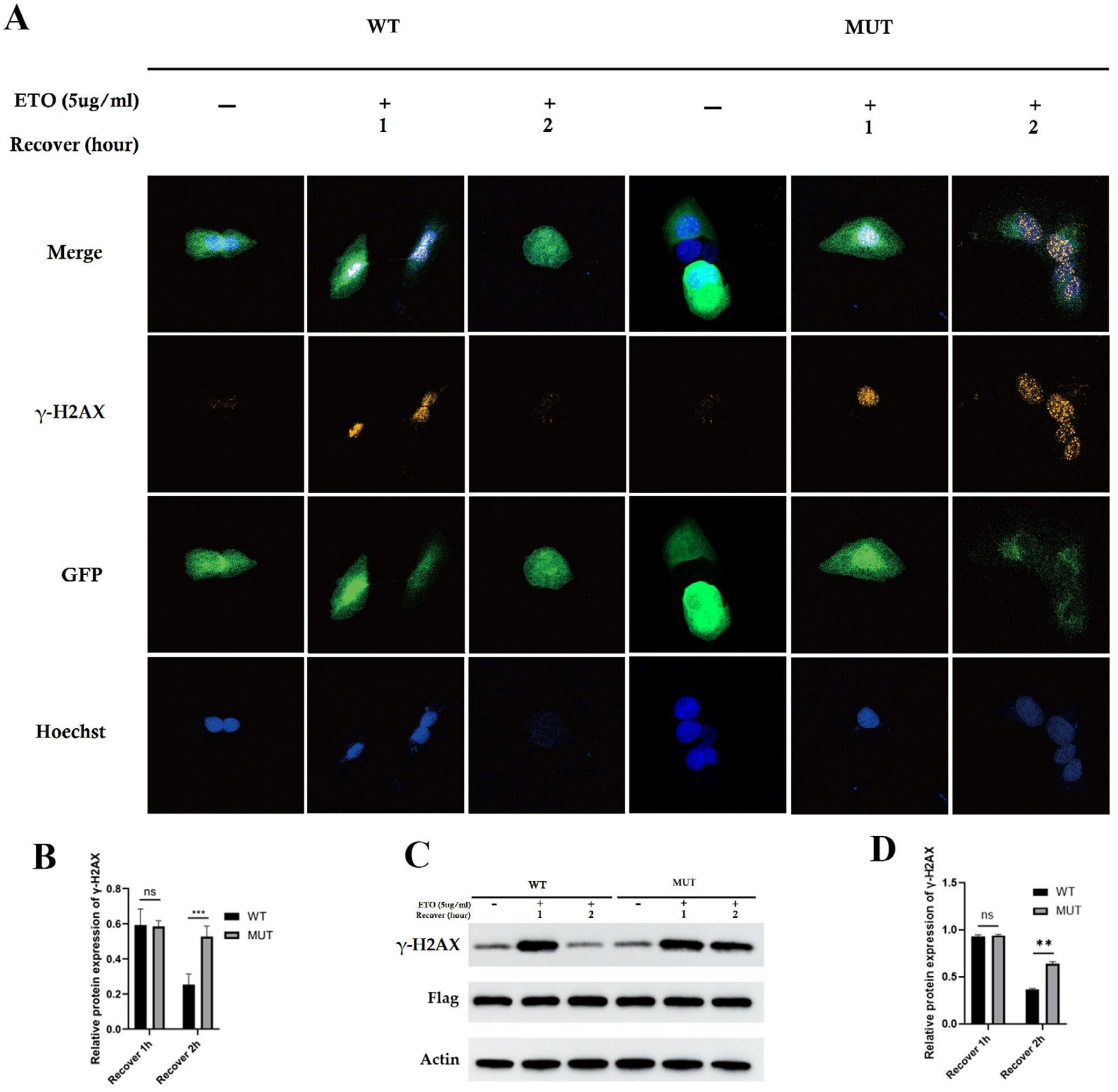
DNA damage and recovery assay. MSH5 c.271 + 1G > A impaired DNA repair. (A, B) Immunofluorescence showed the γ‐H2AX foci formation in U2OS cells overexpressing wild type (WT) or mutant (MUT) MSH5‐GFP protein when suffering from ETO treatment. Scale bars: 5 Lm. (C, D) The γ‐H2AX concentration among U2OS cells wild type (WT) or mutant MSH5‐Flag protein was detected by western blot. The expression of wild type and mutant MSH5 was detected by Flag antibody and β‐Actin was used as the loading control.

In order to understand the effect of this homozygous novel variant (c.271+1G>A) in the MSH5 gene on DNA repair for DSBs, ETO treatment has been given to induce DSBs and observed γ‐H2AX foci have been observed at the DSB sites, which gradually disappeared along with the repair processing (Figure 3A). The result showed that the γ‐H2AX level was much higher in the U2OS cells overexpressing mutant MSH5 than in the wild type (Figure 3A).

Immunofluorescence study showed that compared with the control group, MSH5‐OE, MSH5‐OE‐2, and MSH5‐OE‐3 in the experimental group are the brightest, followed by MSH5‐OE‐1 (Figure 3A,B). It can be seen from the western blot results that, compared with the control group, the γ‐H2AX protein expression level was significantly increased in the MSH5‐OE, MSH5‐OE‐2, and MSH5‐OE‐3 groups, while not significantly increased in the MSH5‐OE‐1 group (Figure 3C,D).

### 3.7 Cell Clone Formation Assay

**FIGURE 4:**
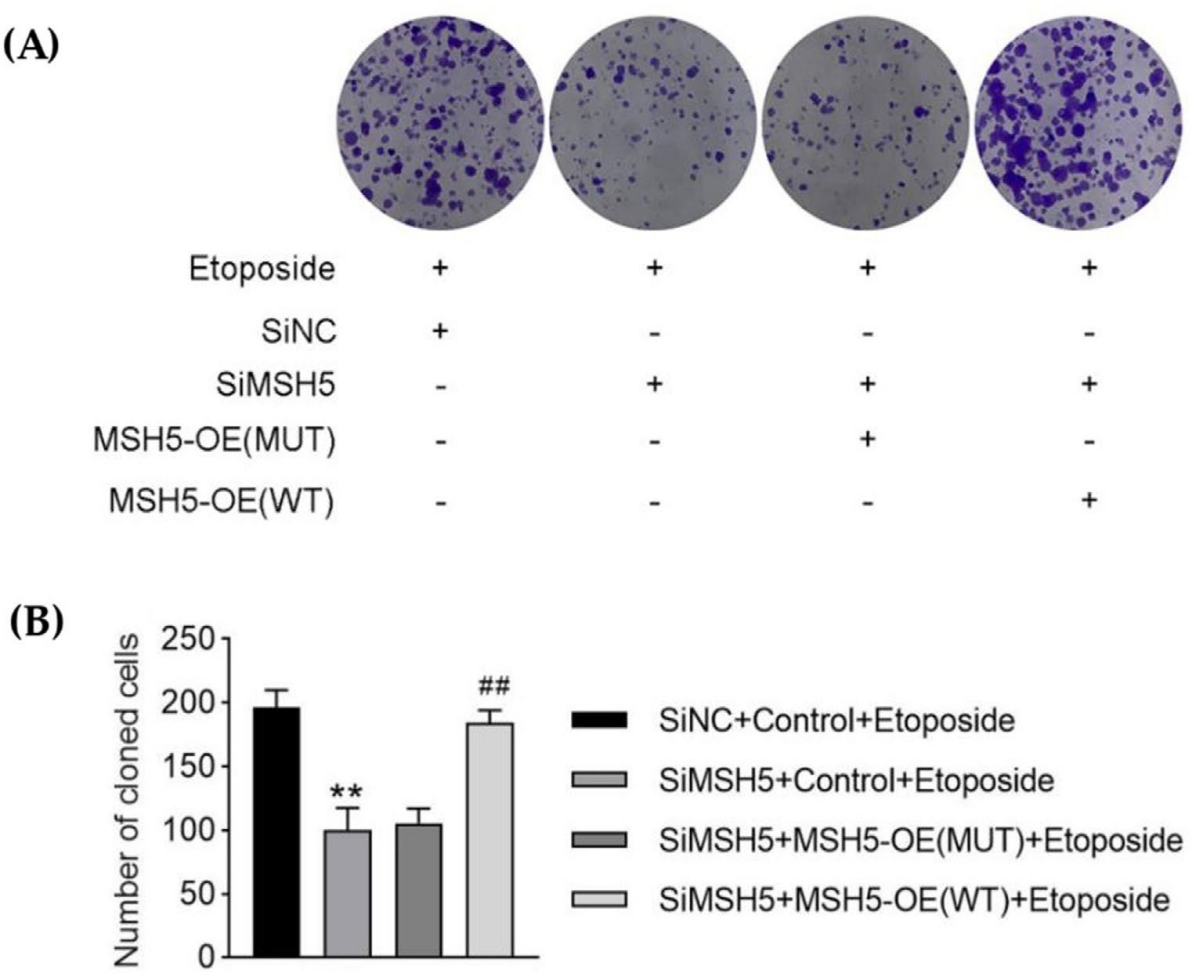
Cell clone formation assay. The cell proliferation capacity of each group was measured by clonal formation assay (**p < 0.01 vs. siNC + control + etoposide group; ##*p* < 0.01 vs. siMSH5 + MSH5‐OE (MUT) + etoposide group). (A, B) showed the clonogenic survival rate of wild type (WT) and mutant (c.271 + 1G > A) overexpressing cells in response to ETO treatment. Cell overexpressing mutant MSH5 showed a lover clonogenic survival rate than wild type. Data in the figure are shown as mean 6 SD. M, siRNA targeting at MSH5; NT, non‐targeting siRNA; WT, wild type.

Our result showed that U2OS cells overexpressing mutant *MSH5* showed a lower clonogenic survival rate than wild type. Hence, this homozygous novel variant (c.271+1G>A) in the *MSH5* gene adversely affects DNA repair capacity or cellular resistance for DSBs (Figure 4A,B).

### 3.8 Cellular Apoptosis Assay

**FIGURE 5:**
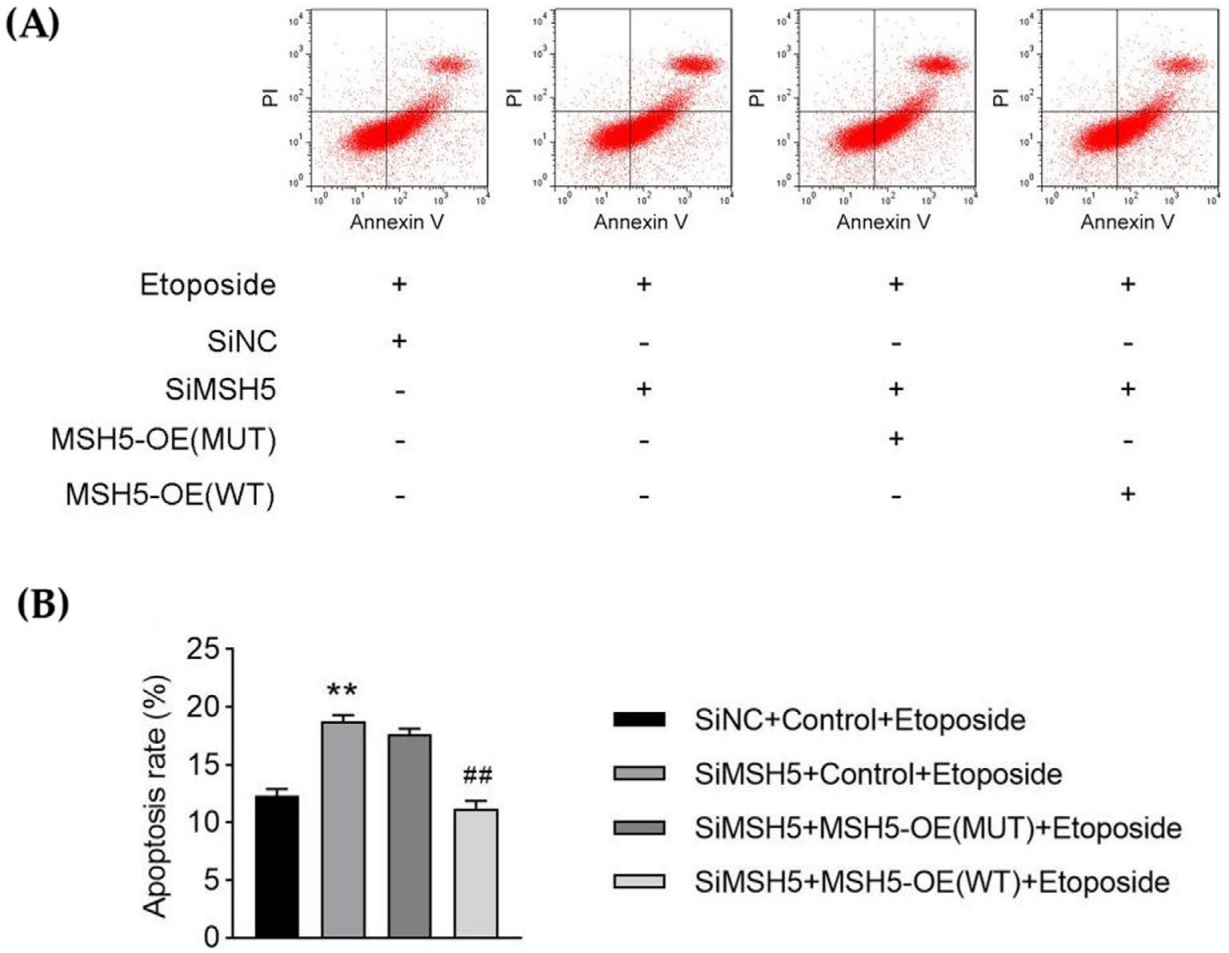
Cellular apoptosis assay. Flow cytometry was used to detect the apoptosis rate in each group (**p < 0.01 vs. siNC + control + etoposide group; ##*p* < 0.01 vs. siMSH5 + MSH5‐OE (MUT) + etoposide group). This novel homozygous mutation causes cellular apoptosis and early cell death. (A, B) Compared with the control group, there was a significant difference in the number of apoptotic cells in these other three experimental groups.

Our result showed that, compared with the control group, there was a significant (*p* < 0.05) difference in the number of apoptotic cells in the mutant group. Hence, this novel homozygous variant causes cellular apoptosis and early cell death (Figure 5A,B).

### 3.9 Cell Counting Kit‐8 (CCK8) Assay

**FIGURE 6:**
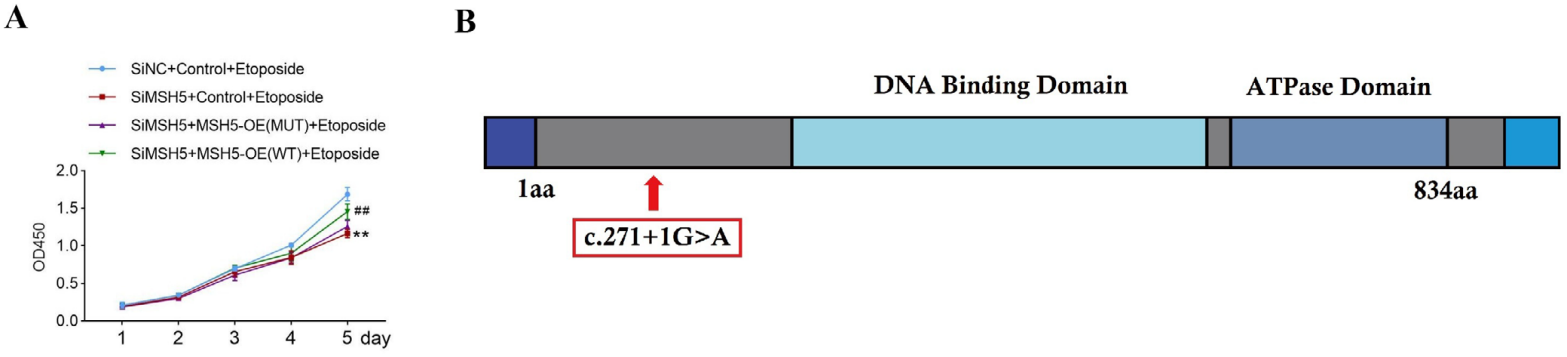
(A) Cell Counting Kit‐8 (CCK8) assay. CCK‐8 assay was used to detect cell viability in each group (**p < 0.01 vs. siNC + control + etoposide group; ##*p* < 0.01 vs. siMSH5 + MSH5‐OE (MUT) + etoposide group). This novel homozygous mutation causes lower cellular proliferation. The CCK8 test results showed that compared with the control group, there was significant increase in cell proliferation in the in siMSH5 + MSH5‐OE (WT) + Etoposide group (*p* < 0.05); while compared with the control group, there was no significant difference in cell proliferation in the siMSH5 + MSH5‐OE (MUT) + Etoposide group (*p* > 0.05). (B) Schematic presentation of MSH5 protein structure. Red box showed the identified mutation (c.271 + 1G > A) in *MSH5* gene in our study. Red arrow denotes the location of mutation.

Our result showed that compared with the control group, there was a significant (*p* < 0.05) difference in the proliferation of cells in the mutant group. Hence, this novel homozygous variant causes lower cellular proliferation (Figure 6A).

## 4 Discussion

In this study, we investigated a Han Chinese family clinically diagnosed with POI. The proband and her two younger sisters were clinically diagnosed with POI. The proband's parents are phenotypically normal. WES identified a homozygous novel splice‐donor site (c.271+1G>A) variant in the *MSH5* gene in this proband. This novel variant was inherited by the proband and her younger sister from their heterozygous parents. This variant causes aberrant splicing of MSH5 mRNA with complete loss of exon 3. Mutant MSH5 mRNA showed significantly reduced expression compared with the wild type transcript. Mutant MSH5 impaired DNA homologous recombination repair and significantly lowered the clonogenic survival rate of cell clones compared with the wild type. This variant also causes a significantly higher apoptotic rate and lower cell proliferation rate than that of the wild type. Moreover, human cells overexpressing mutant MSH5 showed a significantly reduced survival rate and remaining more DSBs unrepaired compared with cells overexpressing wild type MSH5, indicating the impaired function in the process of HR repair. These results suggested that perturbations in MSH5 might not only disturb meiosis in oocytes, but also affect HR repair in granulosa cells during mitosis, followed by accelerated follicle loss through atresia, finally causing POI.

Presently, infertility is an exponentially growing severe reproductive health hazard globally, affecting approximately 10%–15% of couples worldwide [35, 36]. According to the recent report of the Chinese Women and Children Development Center and the China Population Association, the incidence of infertility is 10% in China [37].

Although the majority of infertility cases are caused by abnormalities in male factors, while substantial cases of female infertility are accompanied by POI [36]. Although the age of onset and prevalence of POI is actually uncertain [7]. Moreover, genetic etiologies of POI have been reported with chromosomal aneuploidy, chromosomal microdeletions, and single genetic mutations [3, 38]. However, the majority of POI cases remain unexplained due to phenotypic heterogeneity or overlapping clinical symptoms with ovarian tissue fibrosis and follicular development disorders [2, 16].

Oocyte genome is specifically vulnerable to spontaneous or random occurrence of DNA mismatch damage [39, 40, 41]. During reproduction, DNA damage repair mechanism is playing the key role to maintain the continuous supply of oocytes. DSBs are considered as the most detrimental damage, which is usually repaired by HR and nonhomologous end joining (NHEJ) [42]. MSH4 and MSH5 interact together to form a heterodimeric complex which recognises Holliday junctions followed by the formation of a hydrolysis‐independent sliding clamp that embraces adjacent homologous duplex arms which finally results in HR [43].

Recently, next‐generation sequencing, array‐comparative genomic hybridization (array‐CGH), and single‐nucleotide polymorphism array (SNP‐array) were performed for identifying candidate genes and disease‐causing variants in cohorts of POI patients [13, 15, 44, 45, 46]. Presently, WES is becoming the most potential way of identifying the known as well as the novel candidate genes and their disease‐causing variants underlying POI pathogenesis [17, 47, 48, 49, 50]. WES also enables us to understand the role of these identified candidate genes in ovarian development and POI [51]. WES studies for a large cohort of patients with non‐syndromic POI are very useful for identifying novel candidate genes [9]. However, to date, very few studies were performed with a large cohort of unrelated patients with POI [52, 53, 54]. WES studies performed with single POI pedigrees also identified disease‐causing variants in previously known or novel candidate genes associated with POI [55].

In 2017, Guo et al. investigated a Chinese pedigree with non‐syndromic POI [16]. They performed whole exome sequencing and identified a novel homozygous missense mutation (c.1459G>T, p.Asp487Tyr) in the *MSH5* gene in two sisters with POI. This homozygous mutation impaired DNA homologous recombination repair as well as resulted in atrophic ovaries without oocytes in mice [16]. In 2023, Luo et al. performed next‐generation sequencing of 500 POI patients and identified novel digenic (c.826C>T, p.Arg276Cys in MSH5 gene and c.1063A>G, p.Ile355Val in *MSH4* gene) variants in a Han Chinese woman with non‐syndromic POI [56]. Mandon‐Pépin et al. identified a novel heterozygous mutation p.P29S in the *MSH5* gene in 41 women with POI [57]. In addition, germline variants of the *MSH5* gene were also reported to be associated with nonobstructive azoospermia (NOA) [18, 20, 58, 59]. Till date, only nine germline variants c.1857del, p.Ala620Glnfs* 9, c.1459G>T, p.Asp487Tyr; (c.75dup, p.Ser26Glnfs* 42; c.964C>T, p.Arg322Cys; c.678_681del, p.Tyr227Valfs* 21; c.830C>T, p.Pro277Leu; c.1914C>A, p.Cys638*, c.537+1G>A; c.1126delA, p.Ser376Alafs* 6) of the MSH5 gene have been reported among patients with NOA [18, 20, 58, 59]. Here, we identified the first splice‐site variant in the *MSH5* gene associated with POI (Figure 6B).

In this study, we identified and functionally characterised the disease‐causing variant in the *MSH5* gene in this POI family. However, in future, we would like to perform an in vivo animal model study to understand the effect of this disease‐causing variant of the *MSH5* gene at the phenotypic level. This is the only limitation of our present study. In future, we would also like to recruit a large cohort of patients with POI to understand the genotype–phenotype correlations. In addition, more in‐depth studies in future will help us to attain easy, timely, and accurate clinical diagnosis, therapeutic intervention, treatment, and disease management for the patients with POI.

POI is a very rare genetic reproductive disorder manifested majorly with menstrual disturbances, infertility, and various health problems. Till date, no specific therapeutic strategies have been developed for the patients with POI. POI patients are usually recommended with hormone replacement therapy as the first‐line treatment for POI. Presently, several new therapeutic strategies (in vitro mitochondrial activation, stem cell therapy, exosome therapy, and intra‐ovarian infusion with platelet‐rich plasma) are developing, but none of these emerging therapeutic strategies have yet been standardised for clinical application. Hence, it is a big challenge for the clinical practitioners to select the appropriate therapies for the patients with POI. Here, we applied IVF technologies to overcome the situation of infertility in our studied patient.

## Author Contributions

**Bin Mao:** data curation (equal), formal analysis (equal), visualization (equal), writing – original draft (equal), writing – review and editing (equal). **Lili Zhang:** formal analysis (equal), methodology (equal), writing – original draft (equal), writing – review and editing (equal). **Xiaodong Zhao:** data curation (equal), writing – original draft (equal), writing – review and editing (equal). **Xiaojuan Xu:** data curation (equal), methodology (equal), writing – original draft (equal), writing – review and editing (equal). **Hongfang Liu:** investigation (equal), writing – original draft (equal), writing – review and editing (equal). **Dexiao Song:** formal analysis (equal), visualization (equal), writing – original draft (equal), writing – review and editing (equal). **Peiqiang Li:** formal analysis (equal), investigation (equal), writing – original draft (equal), writing – review and editing (equal). **Santasree Banerjee:** conceptualization (equal), funding acquisition (equal), project administration (equal), supervision (equal), writing – original draft (equal), writing – review and editing (equal). **Xiaoling Ma:** conceptualization (equal), funding acquisition (equal), project administration (equal), supervision (equal), writing – original draft (equal), writing – review and editing (equal). **Yongxiu Yang:** conceptualization (equal), funding acquisition (equal), project administration (equal), supervision (equal), writing – original draft (equal), writing – review and editing (equal).

## Ethics Statement

The present study was approved by the Institutional Review Board of The First School of Clinical Medicine, Lanzhou University, Lanzhou, 730000, China.

## Consent

Written informed consent was obtained from all participating subjects.

## Conflicts of Interest

The authors declare no conflicts of interest.

## Supporting information


**Table S1:** Quality control data of whole exome sequencing for the proband (II‐1).

**Table S2:** Clinical description of the proband (II‐1), proband's younger sister (II‐2) and proband's youngest sister (II‐3).

## Data Availability

All data used for the analyses in this report are available in the CNGB Nucleotide Sequence Archive (CNSA: https://db.cngb.org/cnsa). Accession number: CNP0004873.
