# Supplementary material for: Mutation in MSH5 Causes Primary Ovarian Insufficiency and Successful Therapeutic Intervention by In Vitro Fertilisation
Source: J Cell Mol Med. 2026 May 8;30(9):e70745. doi: 10.1111/jcmm.70745 (PMC13156242; doi:10.1111/jcmm.70745)
Supplement: Supplementary file 1 — Table S1: Quality control data of whole exome sequencing for the proband (II‐1). [file JCMM-30-e70745-s002.docx]

**Supplementary Table S1.** Quality control data of whole exome sequencing for the proband (II-1).

| Total | Raw Reads (All reads) | 319969788 |
| --- | --- | --- |
|  | QC Fail reads | 0 |
|  | Raw Data (Mb) | 15998.49 |
|  | Paired Reads | 319969788 |
|  | Mapped Reads | 317820319 |
|  | Fraction of Mapped Reads | 99.33% |
|  | Mapped Data (Mb) | 15891.02 |
|  | Fraction of Mapped Data (Mb) | 99.33% |
|  | Properly paired | 313387632 |
|  | Fraction of Properly paired | 97.94% |
|  | Read and mate paired | 316892495 |
|  | Fraction of Read and mate paired | 99.04% |
|  | Singletons | 927824 |
|  | Read and mate map to different chromosome | 3080775 |
|  | Read1 | 159984894 |
|  | Read2 | 159984894 |
|  | Read1(rmdup) | 122254358 |
|  | Read2(rmdup) | 122232446 |
|  | forward strand reads | 158910719 |
|  | backward strand reads | 158909600 |
|  | PCR duplicate reads | 73333515 |
|  | Fraction of PCR duplicate reads | 23.07% |
|  | Map quality cutoff value | 20 |
|  | Map Quality above cutoff reads | 287577620 |
|  | Fraction of Map Q reads in all reads | 89.88% |
|  | Fraction of Map Q reads in mapped reads | 90.48% |
| Target | Target Reads | 142976717 |
|  | Fraction of Target Reads in all reads | 44.68% |
|  | Fraction of Target Reads in mapped reads | 44.99% |
|  | Target Data (Mb) | 6522.07 |
|  | Target Data Rmdup (Mb) | 4910.82 |
|  | Fraction of Target Data in all data | 40.77% |
|  | Fraction of Target Data in mapped data | 41.04% |
|  | Len of region | 58682415 |
|  | Average depth | 111.14 |
|  | Average depth (rmdup) | 83.68 |
|  | Coverage (>0x) | 99.75% |
|  | Coverage (>=4x) | 99.55% |
|  | Coverage (>=10x) | 98.61% |
|  | Coverage (>=30x) | 90.32% |
|  | Coverage (>=100x) | 42.46% |
|  | Target Region Count | 199824 |
|  | Region covered > 0x | 199284 |
|  | Fraction Region covered > 0x | 99.73% |
|  | Fraction Region covered >= 4x | 99.49% |
|  | Fraction Region covered >= 10x | 98.61% |
|  | Fraction Region covered >= 30x | 90.50% |
|  | Fraction Region covered >= 100x | 37.93% |
| Flank | Flank size | 200 |
|  | Len of region (not include target region) | 70656846 |
|  | Average depth | 27.71 |
|  | Flank Reads | 54500541 |
|  | Fraction of flank Reads in all reads | 17.03% |
|  | Fraction of flank Reads in mapped reads | 17.15% |
|  | Flank Data (Mb) | 1957.87 |
|  | Fraction of flank Data in all data | 12.24% |
|  | Fraction of flank Data in mapped data | 12.32% |
|  | Coverage (>0x) | 98.61% |
|  | Coverage (>=4x) | 88.32% |
|  | Coverage (>=10x) | 63.36% |
|  | Coverage (>=30x) | 29.52% |
|  | Coverage (>=100x) | 4.28% |
