# Supplementary material for: Mutation in MSH5 Causes Primary Ovarian Insufficiency and Successful Therapeutic Intervention by In Vitro Fertilisation
Source: J Cell Mol Med. 2026 May 8;30(9):e70745. doi: 10.1111/jcmm.70745 (PMC13156242; doi:10.1111/jcmm.70745)
Supplement: Supplementary file 2 — Table S2: Clinical description of the proband (II‐1), proband's younger sister (II‐2) and proband's youngest sister (II‐3). [file JCMM-30-e70745-s001.docx]

**Supplementary Table S2.** Clinical description of the proband (II-1), proband’s younger sister (II-2) and proband’s youngest sister (II-3).

| Test Items | Proband (II-1) | Proband’s younger sister (II-2) | Proband’s youngest sister (II-3) |
| --- | --- | --- | --- |
| Age | 32 years | 31 years | 27 Years |
| Height | 158 cm | 159 cm | 164 cm |
| Body weight | 55 KG | 50 KG | 51.5 KG |
| BMI | 22 | 19.78 | 19.15 |
| Menarche | 16-year-old | 16-year-old | 16-year-old |
| Menstrual cycle | 25-26 Days | 22-23 Days | 30-90 Days |
| Menstrual duration | 6-7 Days | 5-8 Days | 6-7 Days |
| Uterine position | Front position | Front position | Front position |
| Uterine morphology | Normal | Normal | Normal |
| Uterus size  (length, width, and thickness of the uterus) | 80*56*35 cm | 76*50*31 cm | 77*49*33 cm |
| Uterine cavity length | 40 mm | 36 mm | 34 mm |
| Cervical length | 24 mm | 27 mm | 37 mm |
| Endometrial thickness | 29.5 mm | 2 mm | 2 mm |
| Endometrial grading | Menstruation | Menstruation | Menstruation |
| Endometrial echo | Uneven | Uneven | Uneven |
| Right ovary position | Normal | Normal | Normal |
| Right ovarian border | Clear | Not clear | Clear |
| Right ovary size | 19.51*11.35 mm | 17.55*12.88 mm | 15.75*13.27 mm |
| Right follicle diameter (mm) and number | 8.5*1 | No clear small follicles | No clear small follicles |
| Right dominant follicle | No | No | No |
| Left ovary position | Normal | Normal | Normal |
| Left ovarian border | Clear | Not clear | Clear |
| Left ovary size | 21.56*15.81mm | 20.74*12.22 mm | 17.08*8.06 mm |
| Left follicle diameter (mm) and number | 15.5*1 (One)  11.5*1(One) | 2-5*4 (One) | 2*1 (One) |
| Dominant left follicle | 2 | No | No |
| Follicle-forming hormone (FSH) | 19.0mIU/ml | 8.6mIU/ml | 29.1mIU/ml |
| Luteinizing hormone (LH) | 9.3mIU/ml | 7.1mIU/ml | 27.6mIU/ml |
| Progesterone (P) | 0.6ng/ml | 1.21ng/ml | 1.20ng/ml |
| Estradiol (E2) | 38.1pg/ml | 29.3pg/ml | 65.4pg/ml |
| Prolactin (PRL) | 29.3ng/ ml | 11.1ng/ml | 19.1ng/ml |
| Thyrotropin (TSH) | 3.430mU/L | 2.440mU/L | 2.570mU/L |
| Anti-Mullerian hormone (AMH) | 0.507ng/ml | 0.495ng/ml | <0.06ng/ml |
